# Development of a Coal-like Material for Microwave Fracturing: Dielectric Characteristics, Thermal Response, and Mechanical Properties

**DOI:** 10.3390/ma19214548

**Published:** 2026-10-28

**Authors:** Nan Yang, Zhenfan Wang, Guozhong Hu, Haoran Duan, Luchang Xiong, Hongtu Wang, Pan Yang

**Affiliations:** 1 School of Mines, China University of Mining and Technology, Xuzhou 221116, China; 2 School of Safety Engineering, China University of Mining and Technology, Xuzhou 221116, China; 3 Key Laboratory of Xinjiang Coal Resources Green Mining, Ministry of Education, Xinjiang Institute of Engineering, Urumqi 830023, China; 4 Xinjiang Key Laboratory of Coal-bearing Resources Exploration and Exploitation, Xinjiang Institute of Engineering, Urumqi 830023, China; 5 Xinjiang Engineering Research Center of Green Intelligent Coal Mining, Xinjiang Institute of Engineering, Urumqi 830023, China; 6 School of Resources and Safety Engineering, Chongqing University, Chongqing 400044, China

**Keywords:** microwave irradiation, coal-like material, orthogonal experiment, complex permittivity, temperature rise characteristics, mechanical properties

## Abstract

Microwave-induced borehole fracturing of coal seams for pressure relief and permeability enhancement provides a novel approach for preventing coal and gas outburst disasters. However, large-sized natural coal samples suffer from sampling difficulties, complex processing, and poor repeatability, which restrict further related research. In this study, a 2.45 GHz microwave irradiation system with an adjustable power range of 0–2 kW was used, and the specimens were irradiated at a power of 1 kW. Taking bituminous coal as the target, this study used coal powder, river sand, cement, and iron powder to determine and verify the optimal mixing ratio of a coal-like material through orthogonal experiments and multiple linear regression analysis. Taking the imaginary part of the complex permittivity, the temperature non-uniformity coefficient, the uniaxial compressive strength, and the elastic modulus as core indicators, and controlling the error of each indicator relative to the target coal sample within 10%, the optimal ratio was determined as an aggregate-binder ratio of 1.45:1, a coal-to-sand mass ratio of 4:1, and an iron powder mass fraction of 0.75%. The coal-like specimens prepared accordingly showed good consistency with the target coal sample in complex dielectric properties, microwave non-uniform temperature rise characteristics, and mechanical properties, providing a suitable coal-like material for large-scale open-ended microwave borehole fracturing experiments.

## 1. Introduction

As coal mining extends to greater depths, deep coal and rock masses are subjected to complex geological conditions, including high in-situ stress, high ground temperature, and high gas pressure, which significantly increase the difficulty of preventing and controlling coal and gas outburst disasters [1,2]. Artificial active pressure relief, pre-splitting, and permeability enhancement are important technical means to alleviate stress concentration in the coal mass, improve coal seam permeability, and reduce the risk of gas dynamic disasters [3,4,5]. At present, methods such as hydraulic fracturing, pre-splitting blasting, and chemical stimulation have been widely used for coal seam permeability enhancement. Hydraulic fracturing promotes the propagation of coal cracks using high-pressure fluid and can effectively improve coal seam permeability; however, the fracturing fluid tends to cause a water-blocking effect after entering low-permeability coal seams and may result in formation pollution [6]. Pre-splitting blasting breaks coal and rock by the impact generated by blasting, but the strong dynamic disturbance may affect the stability of the surrounding rock of roadways and increase the risk of dynamic disasters [7]. Chemical stimulation mainly improves pore-fracture connectivity by dissolving mineral components with acid solutions, but it suffers from problems such as pipe string corrosion and formation damage [8]. These permeability enhancement methods still have limitations to varying degrees, which restrict their further application in complex deep coal seams. Therefore, it is urgent to explore novel coal and rock permeability enhancement methods with a controllable action range and low pollution.

Microwave irradiation is a non-contact energy input method. Unlike traditional heating methods that rely on heat conduction and convection to achieve temperature rise, microwaves can directly act on the interior of coal and rock masses, converting microwave energy into thermal energy through the dielectric loss of polar molecules in coal and rock, and are characterized by a rapid temperature rise. Coal and rock masses are composed of various minerals, and the dielectric constants and dielectric losses of different mineral components differ to a certain extent. Owing to the selective heating characteristic of microwaves, strongly absorbing minerals are preferentially heated while weakly absorbing minerals lag under microwave irradiation, thereby forming significant temperature gradients and thermal stresses at mineral particle interfaces, inducing intergranular and transgranular microcracks, and ultimately leading to the damage and fracturing of coal and rock [9,10]. Due to the above characteristics, microwave irradiation has become a novel coal and rock fracturing and permeability enhancement method with a controllable action range that is environmentally friendly [11].

Existing studies have mostly carried out microwave irradiation experiments on small-sized standard specimens in closed resonant cavities. Researchers have investigated the damage, fracturing, and pore-permeability structure evolution of coal under microwave irradiation [12,13,14,15], the influence of coal seam bedding on microwave coal breaking [16], the permeability enhancement mechanism of coal under the combined action of external load and microwave irradiation [17,18], the mechanical response of coal under real-time microwave irradiation [19], and the enhancement effect of moisture content on microwave-induced coal fracturing [20,21,22,23]; more recently, the thermal fracturing of anthracite under low-energy microwave irradiation [24] and the damage and seepage characteristics of water-saturated coal under microwave cycling [25] have also been characterized. Another group of researchers has studied the fracture behavior and fracturing mechanism of hard rock under microwave irradiation [26,27,28], which are mainly applied to the physical modification of hard rock by microwaves and assisted TBM rock breaking [29,30,31,32,33,34], as well as the failure mechanisms of rock under microwave treatment [35]. In addition, researchers have established electromagnetic-thermal-mechanical multi-field coupling models for microwave heating of coal and rock [36,37,38] and investigated the enhancement effect of high-dielectric microwave absorbers on microwave coal breaking [39]. Nevertheless, these investigations focus on natural coal or rock, whereas the development of coal-like materials that reproduce the coupled dielectric, thermal, and mechanical responses of coal remains underexplored.

In practical engineering applications, when microwave irradiation of coal and rock is carried out underground, microwaves need to be introduced into the coal seam or rock stratum to heat the coal and rock mass around the borehole. Hu et al. [40] preliminarily investigated the fracturing characteristics of open-ended microwave coal breaking under borehole conditions and established a thermal-mechanical coupling model for open-ended microwave borehole fracturing [41]. However, the size of the coal samples used in their experiments was still small, and the boundary effect was relatively obvious. Meanwhile, the acquisition, transportation, and preparation of large-sized natural coal samples are difficult, and existing studies on coal-like materials mostly focus on the similarity of mechanical and deformation characteristics [42], while research on coal-like materials that can simultaneously satisfy the similarity requirements of complex dielectric properties, microwave non-uniform temperature rise characteristics, and mechanical properties is still lacking. To this end, bituminous coal was selected as the target coal, and a similarity evaluation system for coal-like materials was established based on complex dielectric properties, microwave-induced temperature rise characteristics, and mechanical parameters. Coal powder and river sand were used as aggregates, cement as the binder, and iron powder as an additive to regulate the microwave thermal response. Orthogonal experiments and multiple linear regression analysis were conducted to investigate the effects of the aggregate-to-binder ratio, coal-to-sand mass ratio, and iron powder mass fraction on the similarity indicators. Based on these results, the optimal mix proportions of the coal-like material were determined and experimentally validated, providing a suitable coal-like material for large-scale open-ended microwave borehole fracturing experiments.

## 2. Similarity Evaluation System of Coal-like Material

Under microwave irradiation, the damage evolution of coal generally involves three stages: microwave energy absorption, the development of non-uniform temperature fields and thermal stresses, and the subsequent initiation and propagation of damage and fractures. First, the constituents within the coal absorb microwave energy through dielectric loss, converting electromagnetic energy into thermal energy. Subsequently, differences in the dielectric properties of the various coal constituents result in different heating rates, leading to a pronounced non-uniform temperature distribution and temperature gradients, which in turn generate thermal stresses. Finally, when the thermal stresses exceed the local strength of the coal, the pore–fracture structure is damaged and the mechanical properties progressively deteriorate. Therefore, to ensure that the coal-like material can effectively reproduce the thermal response of the target coal under microwave irradiation, a similarity evaluation system was established based on three aspects: dielectric properties, microwave-induced temperature rise characteristics, and mechanical parameters.

### 2.1. Evaluation Index of Dielectric Properties

The complex permittivity *ε* is commonly used to represent the dielectric properties and polarization characteristics of coal, i.e., the ability of coal to absorb and convert microwaves:

*ε* = *ε^′^* − *jε*^″^ (1)

where *ε*′ is the real part of the complex permittivity, and *ε*″ is the imaginary part of the complex permittivity.

The damage and fracturing effect of coal under microwave irradiation depends on the magnitude of the imaginary part *ε*″ of the complex permittivity, i.e., whether coal can absorb sufficient microwave energy and rapidly convert it into its own thermal energy. Although the real part *ε*′ of the complex permittivity also reflects the polarization response ability of coal to the microwave field, its influence on the thermal response of microwaves and the damage and fracturing process of coal is weaker than that of the imaginary part. Therefore, this study selects the imaginary part *ε*″ of the complex permittivity of the coal-like material as the core evaluation index, and the real part *ε*′ of the complex permittivity as the verification index.

### 2.2. Evaluation Index of Temperature Rise Characteristics

Under microwave irradiation, coal not only exhibits an overall temperature rise but also shows a non-uniform temperature distribution. It is difficult to accurately describe the microwave thermal response characteristics of the coal-like material using only a single temperature parameter. To this end, this study selects the average temperature *T_avg_* and the temperature non-uniformity coefficient *R_M_*_/*A*_ to evaluate the temperature rise characteristics of the coal-like material under microwave irradiation:

(2) $$R_{M/A}=\frac{T_{\max}}{T_{\mathrm{avg}}}$$

where *T_max_* is the maximum temperature of the material; *T_avg_* is the average temperature of the material, and *R_M_*_/*A*_ is the temperature non-uniformity coefficient, which represents the non-uniformity of the temperature of the coal-like material.

Unlike conventional heating, coal under microwave irradiation is not simply uniformly heated but undergoes a non-uniform and complex heating process involving local hot spots and temperature gradients. If only the average temperature of the coal-like material under microwave irradiation is considered, it is very likely that the overall temperature rise in coal is similar while the local hot spot characteristics are completely different. In this case, even if the average temperature of the coal-like material is close to that of the target coal sample, its damage and fracturing effect will still differ significantly. Therefore, this study takes the temperature non-uniformity coefficient *R_M_*_/*A*_ as the core evaluation index and the average temperature *T_avg_* as the verification index.

### 2.3. Evaluation Index of Mechanical Parameters

The coal-like material also needs to have a certain similarity with the target coal sample in terms of mechanical parameters, to ensure that its damage evolution and crack propagation under microwave irradiation have characteristics similar to those of the target coal sample. This study selects three parameters, namely the uniaxial compressive strength *R_c_*, the elastic modulus *E*, and the tensile strength *R_t_*, as the mechanical parameter evaluation indexes.

The similarity of the uniaxial compressive strength *R_c_* to that of the target coal sample is not sufficient to guarantee the similarity in mechanical properties. If the elastic modulus *E* differs greatly, the thermal stress evolution, crack initiation and propagation, and failure mode under microwave irradiation may still be different. Therefore, this study takes the uniaxial compressive strength *R_c_* and the elastic modulus *E* as the core indexes, and the tensile strength *R_t_* as the verification index.

## 3. Preparation of Coal-like Material

### 3.1. Characteristic Parameters of the Target Coal Sample

**Figure 1:**
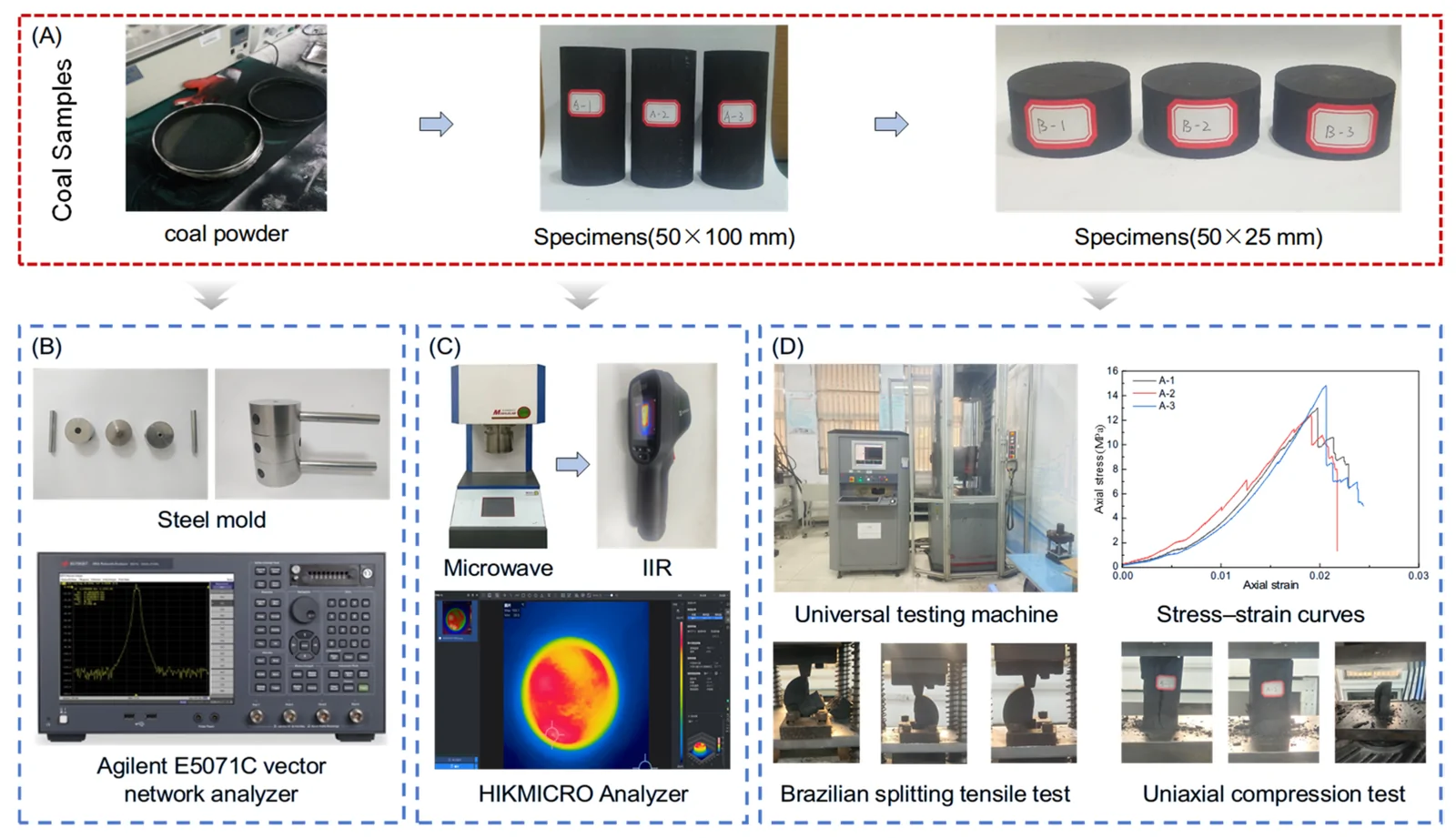
Characterization methods for target coal samples: (**A**) coal sample preparation; (**B**) complex permittivity measurement; (**C**) microwave irradiation and surface temperature monitoring; (**D**) mechanical property testing.

In this experiment, bituminous coal from a mine in Inner Mongolia was selected as the target coal sample. To obtain the mechanical characteristic parameters of the target coal sample and its temperature rise behavior under microwave irradiation, large blocks of bituminous coal were prepared into two types of standard cylindrical specimens with diameters of φ50 mm × 100 mm and φ50 mm × 25 mm by drilling and cutting. The non-parallelism of the two end faces of all specimens was less than 0.05 mm, as shown in Figure 1A.

#### 3.1.1. Dielectric Parameters

The transmission/reflection method is a commonly used method for measuring the dielectric parameters of materials, with advantages such as convenient operation, high accuracy, strong applicability, and low sample consumption. In this study, an Agilent E5071C vector network analyzer was used to measure the complex permittivity of the target coal sample and the coal-like material, as shown in Figure 1B. To meet the shape requirements of the vector network analyzer for the test specimen, raw coal powder was thoroughly mixed with molten paraffin at a volume ratio of 4:3 in a constant-temperature water bath at 20 °C, and the mixture was then pressed into ring-shaped specimens with an outer diameter of 7 ± 0.2 mm, an inner diameter of 3.04 mm, and a thickness of 2.0 mm using a specially designed mold.

When calculating the dielectric parameters, it is assumed that the coal powder and paraffin are uniformly mixed, and the complex permittivity of the specimen to be measured can be expressed as:

ln*ε_m_* = *v_c_*ln*ε_c_* + *v_p_*ln*ε_p_* (3)

where *ε_c_* is the complex permittivity of coal, *ε_p_* is the complex permittivity of paraffin, *v_c_* is the volume fraction of coal (taken as 4/7), and *v_p_* is the volume fraction of paraffin (taken as 3/7). The test results show that, at a microwave frequency of 2.45 GHz, the real part of the complex permittivity of the target coal sample ranges from 3.369 to 3.562 with a mean value of 3.474, and the imaginary part ranges from 0.328 to 0.352 with a mean value of 0.336.

#### 3.1.2. Temperature Rise Parameters

In this study, a self-developed microwave irradiation apparatus was used to test the temperature rise characteristics of the target coal sample under microwave irradiation, as shown in Figure 1C. The microwave apparatus mainly consists of a high-voltage converter, a microwave generator, a waveguide, an antenna, a resonant cavity, an exhaust port, an air-cooling system, and a control system, with a microwave frequency of 2.45 GHz and a microwave power adjustment range of 0–2 kW. In addition, the microwave resonant cavity is a cylindrical sealed metal cavity of φ150 mm × 300 mm to ensure the safety of the experiment.

**Figure 2:**
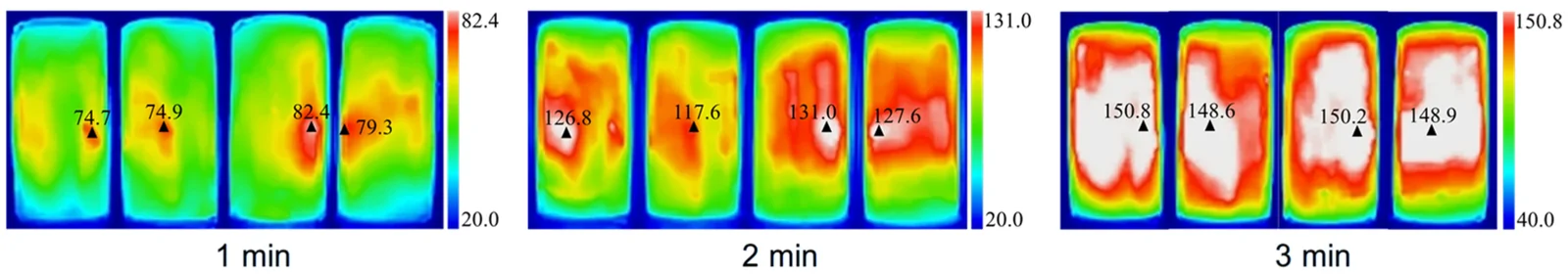
Infrared thermal images of the target coal sample under 1 kW microwave irradiation for 1, 2, and 3 min.

In this study, the target coal sample was subjected to microwave treatment at a microwave power of 1 kW for an irradiation time of 3 min. The sample was taken out every 1 min, and infrared thermal images were captured using a HIKMICRO infrared thermal imager. The experimental results are shown in Figure 2. It can be seen from Figure 2 that the surface temperature field of the target coal sample exhibits an obvious non-uniform distribution under microwave irradiation. After 1 min of microwave irradiation, a local high-temperature zone had already appeared on the surface of the target coal sample, with a maximum temperature of 74.0–82.4 °C, whereas the temperature in the edge region was relatively low. After 2 min, the overall temperature of the target coal sample increased significantly, with the maximum temperature rising to 117.6–131.0 °C; the hot zone continued to expand, and the non-uniformity of the surface temperature field was further enhanced. After 3 min, the maximum temperature of the target coal sample further increased to 148.6–150.8 °C, and a larger continuous high-temperature zone had formed, although local low-temperature zones still existed. This indicates that in the later stage of microwave irradiation, the overall temperature rise level of the target coal sample was further improved, but the surface temperature field still exhibited an obvious non-uniform distribution. Table 1 presents the temperature parameters of the target coal sample after 3 min of microwave irradiation.

#### 3.1.3. Mechanical Parameters

In this study, an electro-hydraulic servo universal testing machine, as shown in Figure 1D, was used to test the uniaxial compressive strength and elastic modulus of the target coal sample. During the experiment, force loading was performed under displacement control at a loading rate of 0.3 mm/min until the coal sample failed. It can be seen from the failure morphology of the target coal sample after uniaxial compression in Figure 1D that the failure of the coal sample was dominated by vertical splitting, and obvious vertical main cracks appeared on the surface of the sample, penetrating most of the specimen area. Based on the stress-strain curve of the target coal sample, the uniaxial compressive strength and elastic modulus were calculated, and the experimental results are shown in Table 2.

In addition, the Brazilian splitting method was used to test the tensile strength of the target coal sample. To accurately measure the tensile strength, the arc-shaped loading block method recommended by the International Society for Rock Mechanics (ISRM) was adopted. The failure morphology of the target coal sample in the tensile test is shown in Figure 1D.

### 3.2. Preparation Scheme and Process of the Coal-like Material

Similarity criteria are an important basis for the selection of raw materials for similar materials. However, it is often difficult to satisfy all similarity conditions simultaneously in practical research. Therefore, the similar material adopted in the laboratory should possess the following characteristics: (i) the physical properties of the raw materials used to prepare the coal-like material should be relatively stable, so as to facilitate quantitative control; (ii) the raw materials should be non-toxic and harmless; (iii) the raw materials should have wide sources, low cost, and be convenient for batch preparation; (iv) the coal-like material should exhibit good repeatability in terms of dielectric properties, mechanical properties, and thermal response characteristics; and (v) the preparation process of the coal-like material should not be overly complex, so that it can be prepared in large quantities within a short time.

#### 3.2.1. Selection of Raw Materials

The preparation of similar materials generally involves mixing raw materials such as aggregate, binder, additive, and water uniformly in a certain proportion, followed by curing and shaping in a mold. Therefore, different types and proportions of raw materials all influence the dielectric properties, microwave-heating temperature rise characteristics, and mechanical properties of the similar material.

(1) Aggregate.

In existing studies on coal similar materials, coal powder, river sand, or a combination of the two are common aggregates. Coal powder is the closest to raw coal in composition and basic properties, which can improve the similarity between the similar material and the target coal sample in terms of dielectric properties and thermal response; river sand has the advantages of wide sources, stable properties, and low cost, and can be used to adjust the mechanical properties and deformation characteristics of the similar material.

In addition, the particle sizes of coal powder and river sand also have an important influence on the stability of the similar material. Considering the forming and strength requirements of the similar material, the aggregate in this study was composed of coal powder with two particle sizes of 10–20 mesh and 20–35 mesh mixed at a ratio of 1:1, while the particle size of river sand was 10–70 mesh.

(2) Binder.

At present, the commonly used binders in coal similar materials include cement, sodium humate, gypsum, and others, among which cement is widely applied in engineering model tests and studies on coal-rock similar materials owing to its wide sources and mature sample-preparation process. After hydration, cement can form a stable cementation structure between coal powder and river sand particles, and the strength of the similar material is positively correlated with the content of the binder.

(3) Additive.

Coal exhibits an obvious non-uniform heating characteristic under microwave irradiation, and the high thermal stress formed by local hot spots in coal is often the key to the evolution of the pore-fracture structure. Therefore, in addition to ensuring that the similar material possesses dielectric and mechanical properties similar to those of the target coal mass, it should also exhibit non-uniform thermal response characteristics similar to those of coal under microwave irradiation. In this study, copper powder, steel slag powder, pyrite powder, iron powder, Fe_3_O_4_ powder, and Fe_2_O_3_ powder were selected as candidate additives, and comparative microwave heating tests were carried out on different coal powder-additive mixtures according to the scheme in Table 3. The temperature of the mixture was recorded every 20 s during the test to ultimately screen out the suitable additive.

**Figure 3:**
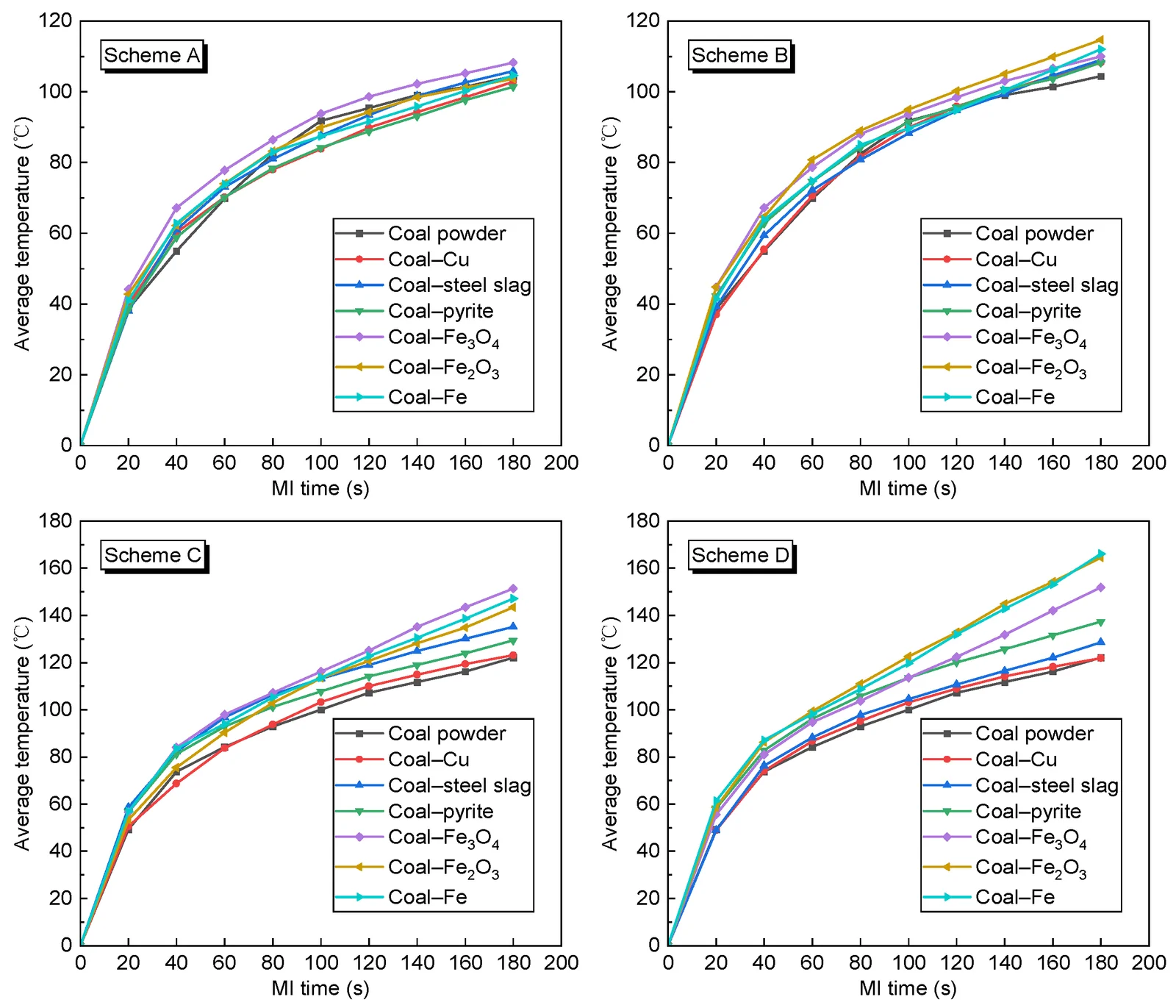
Average temperature rise curves of the mixtures with different additives under microwave irradiation.

Figure 3 shows the average temperature rise curves of the mixtures under microwave irradiation for each scheme. It can be seen from the figure that all types of additives can improve the average temperature rise in the mixture to a certain extent, indicating that the additives can effectively improve the microwave thermal response ability of the mixture, thereby promoting the formation of hot spots in coal. Among them, iron powder, Fe_2_O_3_ powder, and Fe_3_O_4_ powder can significantly promote the temperature rise in the mixture, followed by pyrite powder, whereas the enhancement effect of copper powder and steel slag powder is relatively limited. The marked differences among the additives can be attributed to their distinct dielectric loss properties: iron powder and the iron oxides (Fe_2_O_3_ and Fe_3_O_4_) exhibit strong magnetic and dielectric losses, which enable them to absorb microwave energy efficiently and convert it into heat, whereas copper powder and steel slag powder has a lower microwave-absorbing capacity, so their heating enhancement is limited. In addition, within the tested range, the enhancement effect increases with the additive dosage, because a higher content of microwave-absorbing particles provides more localized heat sources that promote hot-spot formation and accelerate the overall temperature rise in the mixture.

**Figure 4:**
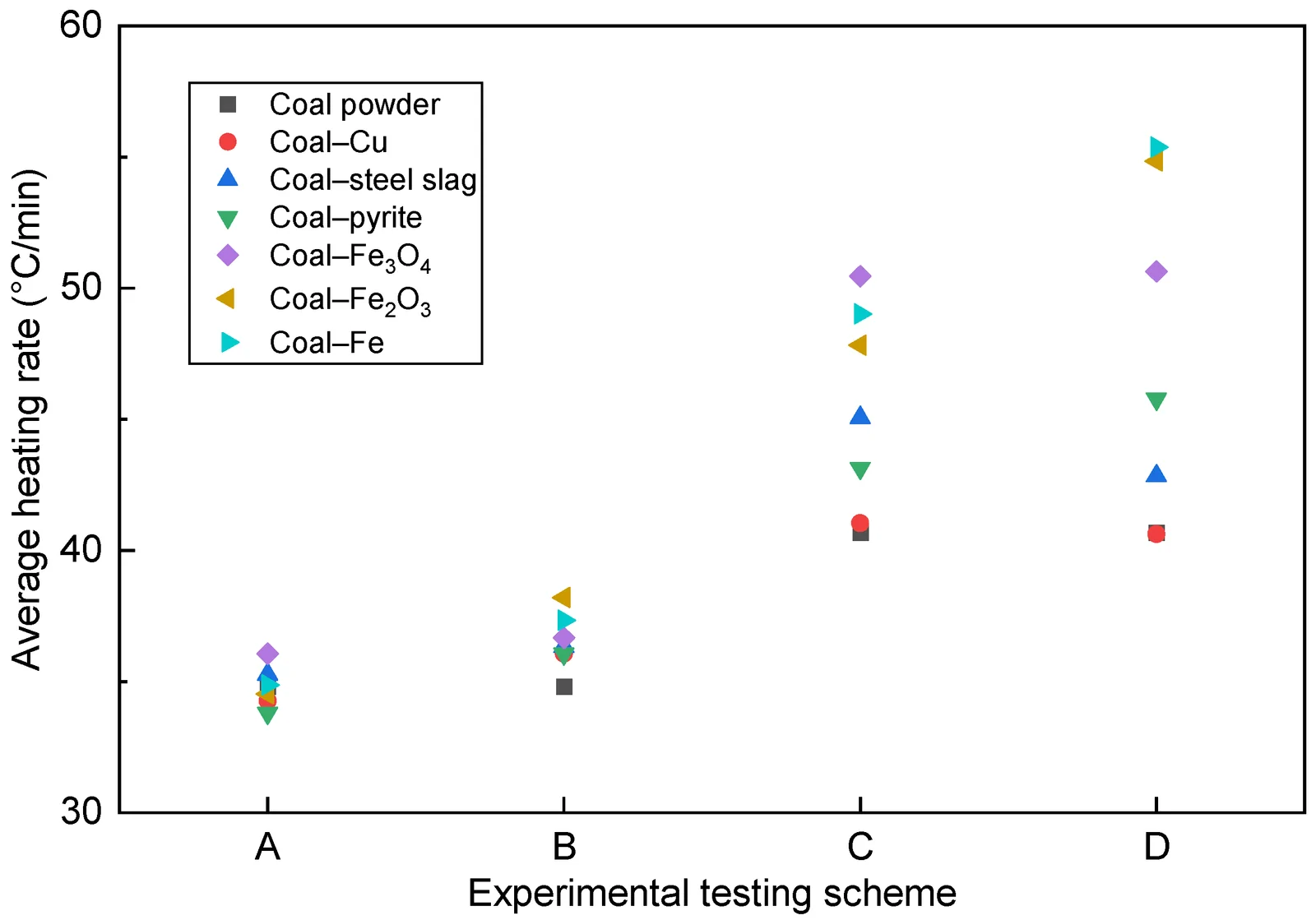
Average temperature rises rates of the mixtures with different additives under microwave irradiation.

In this study, the content of the additive was relatively low, and its main role was not to change the overall dielectric properties of the similar material, but to promote the formation of local hot spots in coal and enhance the non-uniform temperature rise in the coal mass, thereby improving the similarity of the microwave thermal response characteristics between the similar material and the real coal mass. From the average temperature rise rates of each additive in Figure 4, iron powder not only exhibited a higher temperature rise rate under different microwave powers and different addition ratios, but its temperature rise rate was also more stable than those of Fe_2_O_3_ powder and Fe_3_O_4_ powder. Therefore, iron powder was selected as the additive of the similar material in this study.

(4) Water content.

In the preparation process of the coal-like material, cement undergoes a hydration reaction after mixing with water, forming a cementation structure between particles such as coal powder and river sand, thereby improving the cementation performance and mechanical strength of the similar material. However, the water content needs to be controlled within an appropriate range. When the water content is too low, the cement hydration reaction is insufficient and the materials are not mixed uniformly, which can easily cause cracks or even fracture during specimen demolding. In contrast, when the water content is too high, the specimen is prone to water seepage and stratification during preparation, which affects the uniformity and overall performance of the similar material.

#### 3.2.2. Experimental Scheme

The preparation process of the coal-like material involves multiple factors, and there are certain interactive effects among these factors. Therefore, it is necessary to design different experimental combinations for multiple factors and multiple levels to carry out orthogonal tests, to study the influence of raw materials such as coal powder, river sand, cement, and iron powder on the dielectric properties, microwave-heating temperature rise characteristics, and mechanical properties of the coal-like material. The orthogonal test in this study was designed with three factors, namely the aggregate-binder ratio A (*m_aggregate_*:*m_binder_*), the coal-to-sand mass ratio B in the aggregate (*m_coal powder_*:*m_river sand_*), and the iron powder mass fraction C (*m_iron powder_*). Four levels were set for each factor, among which the aggregate-binder ratio was 2:1, 3:2, 1:1, and 2:3; the coal-to-sand mass ratio was 4:1, 2:1, 1:1, and 1:2; and the iron powder mass fraction was 0%, 0.5%, 1.0%, and 2.0%. During the test, the water-cement ratio was controlled at 0.35, and the specific scheme is shown in Table 4 and Table 5.

After the tests were completed, the orthogonal test results were mainly processed using three methods: range analysis, variance analysis, and regression analysis.

(1) Range analysis. The range is the difference R between the maximum and minimum values in the same group of data. A larger range R indicates that the test index is more sensitive to that factor; therefore, the primary and secondary order of the influence of each factor on the test results can be judged by comparing the magnitude of the range R. However, this analysis method has certain limitations in the determination of test errors.

(2) Variance analysis. Variance analysis can separate the changes in factors and levels from the test error, and judge from a statistical perspective whether each factor has a significant influence on the test results. In variance analysis, the P value represents the degree to which the factor does not produce a significant influence on the test results. When *P* ≤ 0.01, the influence of each factor on the evaluation index is considered extremely significant; when 0.01 ≤ *P* ≤ 0.05, the influence of each factor on the evaluation index is considered significant; when *P* ≥ 0.05, the influence of each factor on the evaluation index is considered insignificant.

(3) Regression analysis. Regression analysis is used to establish the quantitative relationship between each response index and the proportioning factors. To ensure the consistency of the regression analysis method for different evaluation indexes, this study first established a multiple linear regression model containing all proportioning factors, and then simplified the linear regression model by comprehensively considering the results of variance analysis, the significance of the regression coefficients, and the physical significance of the proportioning factors.

#### 3.2.3. Preparation Process

**Figure 5:**
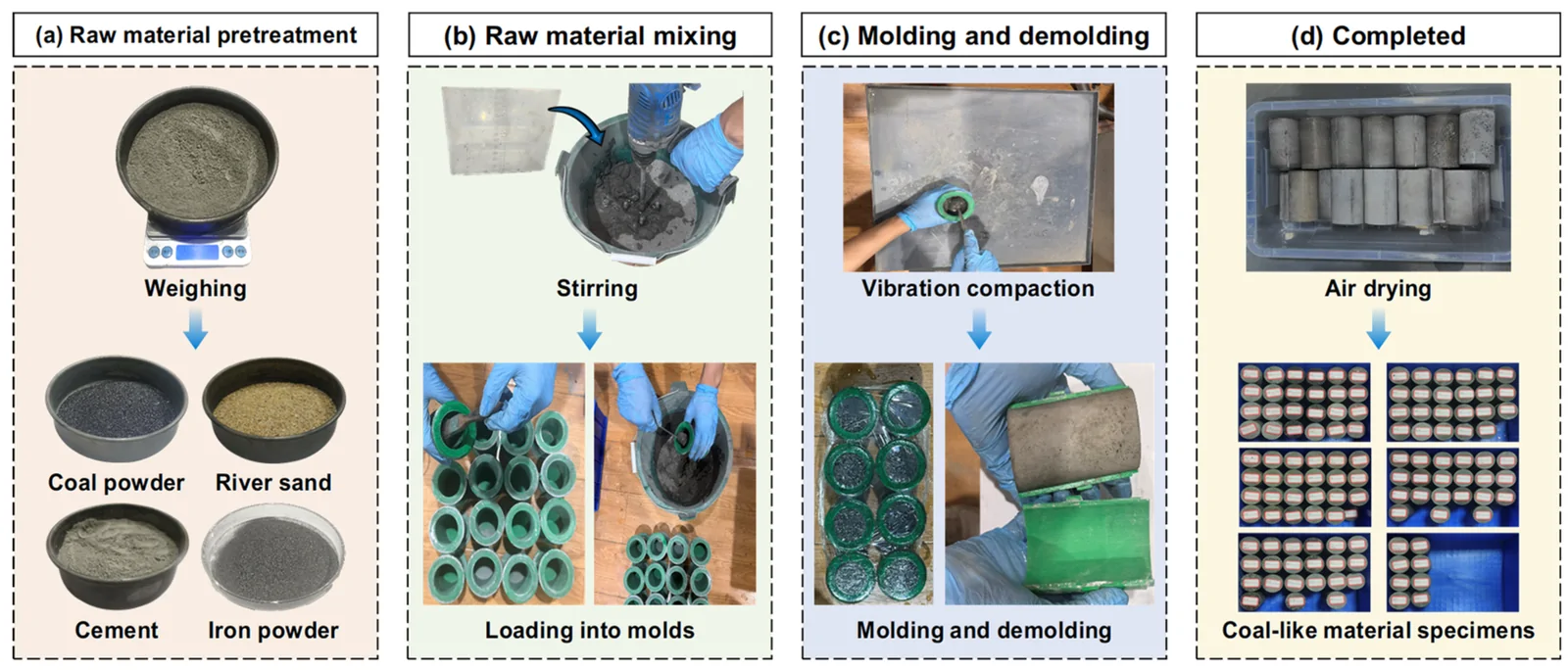
Preparation process of the coal-like specimens: (**a**) raw material pretreatment; (**b**) raw material mixing; (**c**) molding and demolding; (**d**) completed specimens.

The raw materials of the coal-like material in this study mainly include coal powder, river sand, cement, and iron powder. During the preparation process, equipment such as molds, mixers, and vibrating tables are also required, as shown in Figure 5. The specific steps are as follows:

(1) Weighing of raw materials. According to the proportioning determined by the orthogonal test scheme in Table 5, raw materials such as coal powder, river sand, cement, iron powder, and water were accurately weighed. To ensure the accuracy of the proportioning, a high-precision electronic scale was used for weighing, and the dosage error of each material was strictly controlled.

(2) Mixing and stirring of raw materials. The weighed coal powder, river sand, cement, and iron powder were added to the mixing container in sequence, and dry mixing was first carried out to make all raw materials fully and uniformly mixed. Subsequently, the required amount of water was slowly added according to the designed proportion, and stirring was continued. The stirring time was generally not less than 4 min, so as to avoid phenomena such as agglomeration, stratification, or insufficient local stirring.

(3) Oil coating of the mold. Before loading, the inner wall of the mold was uniformly coated with a release oil to reduce the adhesion between the specimen and the mold and avoid specimen damage during the demolding process.

(4) Loading and vibration. The uniformly stirred mixture was loaded into the mold by layered loading and synchronous vibration, to reduce the internal pores of the specimen while avoiding phenomena such as aggregate sinking and slurry stratification caused by excessively long vibration time.

(5) Demolding and curing. After loading, the mold was placed in a constant-temperature and constant-humidity environment for 24 h to complete preliminary setting and forming. After 24 h of setting and forming, the specimen was demolded, and the demolded specimen was placed in a curing box for 7 d to ensure that the similar material underwent sufficient hydration reaction and to promote the development of the cementation structure inside the specimen. After curing, the specimen was placed in a ventilated environment for further air drying for 28 d, to meet the subsequent testing requirements for dielectric parameters, microwave-heating temperature rise parameters, and mechanical parameters.

Both the pre-treatment of the raw materials and the mixing speed influence the final properties of the coal-like specimens. In this study, the coal powder and river sand were screened to the specified particle-size ranges before mixing to ensure uniform grading, and dry mixing was performed before water addition to avoid agglomeration. The mixing speed and duration were controlled (not less than 4 min at a moderate speed), because insufficient mixing leads to a local concentration of the binder or additive and thus to non-uniform mechanical and dielectric properties, whereas excessive or over-rapid mixing can entrain air and cause aggregate segregation. These controls are essential for the repeatability of the prepared specimens.

## 4. Results

### 4.1. Sensitivity Analysis of Factors Affecting the Similarity Performance of Coal-like Materials

#### 4.1.1. Dielectric Similarity

(1) Range analysis of complex permittivity

**Figure 6:**
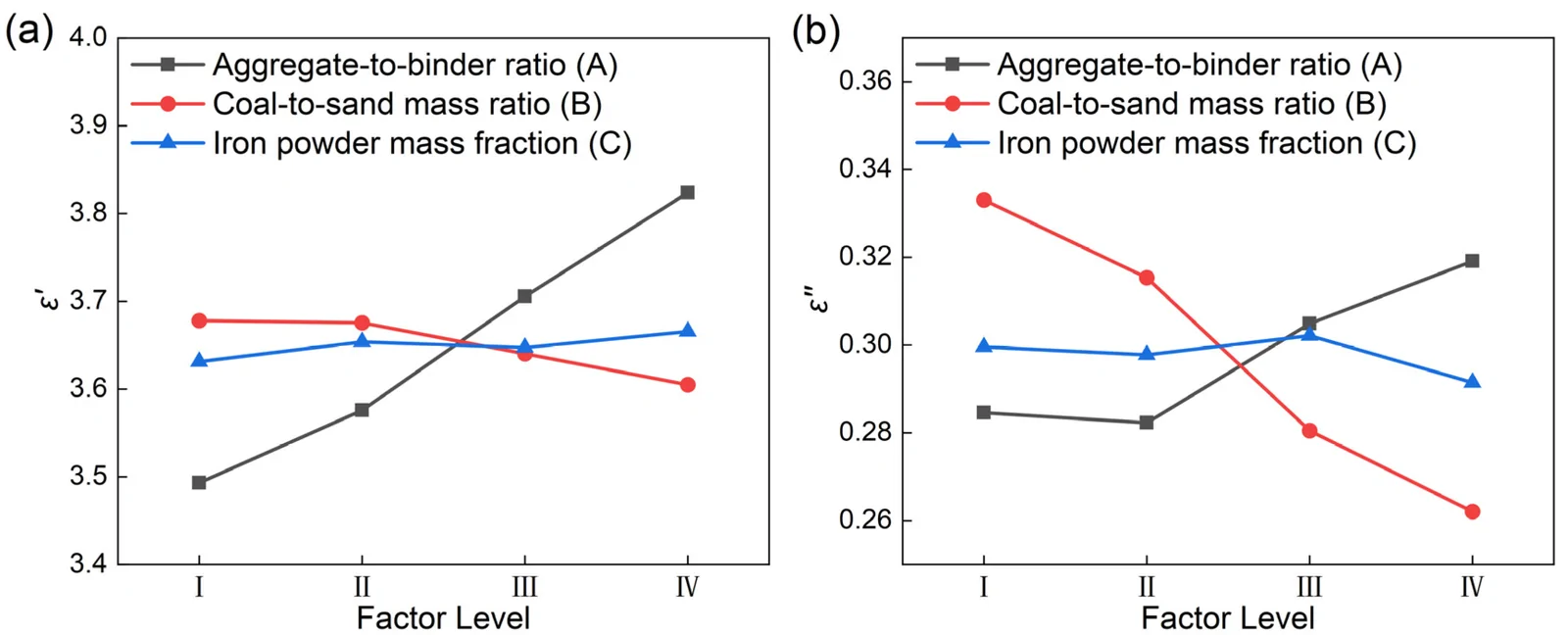
Sensitivity curves of the factors affecting the complex permittivity of coal-like specimens: (**a**) real part *ε*′; (**b**) imaginary part *ε*″.

Based on the test results of the real and imaginary parts of the complex permittivity of the specimens in Table 6, the mean values and ranges of the real and imaginary parts of the complex permittivity under different factors and levels were calculated, as shown in Figure 6.

As shown in Figure 6a, the ranges of the real part *ε*′ of the complex permittivity of the specimens under factors A, B, and C are 0.331, 0.073, and 0.034, respectively, and the order of influence of each factor on the real part *ε*′ of the complex permittivity is: *R*_A_ > *R*_B_ > *R*_C_. In addition, as the aggregate-binder ratio (A) decreases, the mean value of the real part *ε*′ of the complex permittivity gradually increases from 3.493 to 3.824, showing an overall upward trend; as the coal-to-sand mass ratio (B) decreases, the real part *ε*′ of the complex permittivity generally shows a downward trend, but with a relatively small variation range; as the iron powder mass fraction (C) increases, the variation in the real part *ε*′ of the complex permittivity is not obvious. The aggregate-binder ratio has the best regulating effect on the real part *ε*′ of the complex permittivity, while the regulating effects of the coal-to-sand mass ratio and the iron powder mass fraction are relatively limited.

Unlike the real part, the sensitivity order of the imaginary part *ε*″ of the complex permittivity to factors A, B, and C is *R*_B_ > *R*_A_ > *R*_C_, with ranges of 0.071, 0.037, and 0.011, respectively. As the coal-to-sand mass ratio decreases, the mean value of the imaginary part *ε*″ continuously decreases from 0.333 to 0.262, showing the largest variation; a change in the aggregate-binder ratio causes the imaginary part *ε*″ to first decrease slightly and then increase significantly; under different iron powder mass fractions, the imaginary part *ε*″ of the complex permittivity changes little. Therefore, the coal-to-sand mass ratio has the most significant influence on the imaginary part *ε*″ of the complex permittivity.

(2) Variance analysis of complex permittivity

To further examine the influence of each factor on the complex permittivity of the specimens, the variances of the real and imaginary parts of the complex permittivity were calculated, and the results are shown in Table 7.

For the real part *ε*′ of the complex permittivity, the P-value of the aggregate-binder ratio (A) is <0.001, indicating an extremely significant influence on the real part of the complex permittivity, whereas the P-values of the coal-to-sand mass ratio (B) and the iron powder mass fraction (C) are 0.153 and 0.738, respectively, both indicating insignificant influences. For the imaginary part *ε*″ of the complex permittivity, the P-value of the coal-to-sand mass ratio (B) is <0.001, indicating an extremely significant influence, and the aggregate-binder ratio (A) also has an extremely significant influence with a P-value of 0.002; the P-value of the iron powder mass fraction (C) is 0.376, indicating an insignificant influence.

From the above analysis, the dominant factor for the real part *ε*′ of the complex permittivity is the aggregate-binder ratio, while the dominant factor for the imaginary part *ε*″ is the coal-to-sand mass ratio. Therefore, in the adjustment and optimization of the mix proportion of the coal-like material, the aggregate-binder ratio and the coal-to-sand mass ratio should be coordinated and controlled, while the iron powder mass fraction serves as an additive regulating parameter.

#### 4.1.2. Similarity of Microwave Temperature Rise Characteristics

The temperature rise characteristics can reflect not only the ability of the similar material to absorb and convert microwave energy but also the distribution of the surface temperature of the specimen, and are an important basis for judging whether the coal-like material can effectively simulate the microwave response characteristics of the target coal sample. In this study, the prepared coal-like specimens were subjected to microwave irradiation heating at a microwave power of 1 kW for 3 min. The specimens were taken out every 1 min, and infrared thermal images were captured using a HIKMICRO infrared thermal imager.

**Figure 7:**
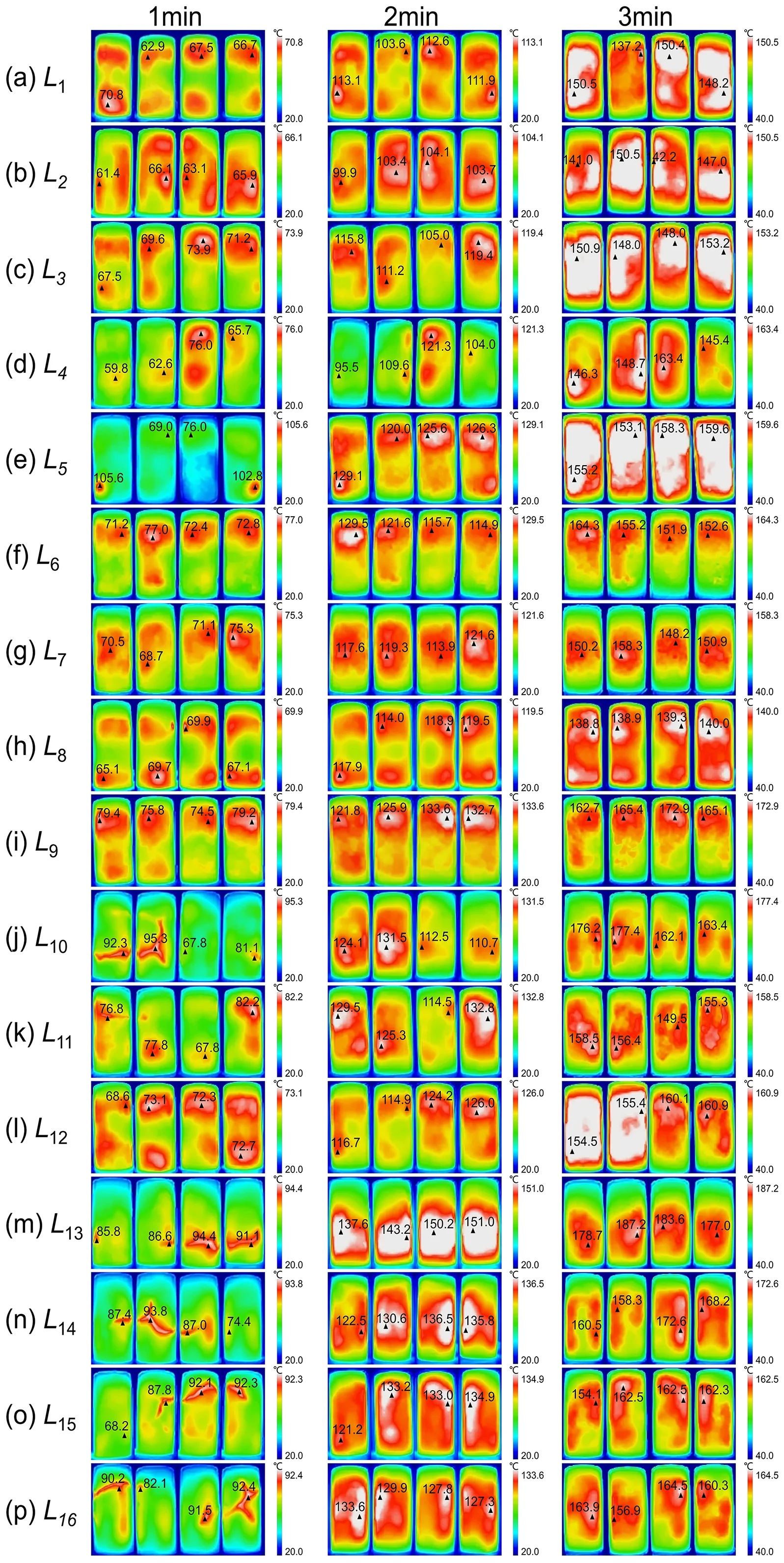
Infrared thermal images of coal-like specimens (**a**–**p**) under different microwave irradiation times.

Figure 7 shows the surface temperature field distribution characteristics of the 16 groups of coal-like specimens under microwave irradiation for 1 min, 2 min, and 3 min. The L_2_, L_6_, L_7_, L_9_, L_11_, L_15_, and L_16_ specimens all exhibited the variation characteristic of preferential temperature rise in the upper-middle region, followed by local expansion and finally the formation of a stable high-temperature zone, and their temperature evolution was closest to that of the target coal sample. The L_1_, L_3_, L_8_, and L_12_ specimens showed multiple dispersed hot spots after 1 min of microwave irradiation, and these hot spots gradually merged into a large high-temperature zone with increasing irradiation time. The L_4_, L_5_, and L_13_ specimens exhibited slow temperature rise in the early stage and accelerated temperature rise in the later stage, which differs to some extent from the relatively continuous and progressive thermal response process of the target coal sample. The L_10_ and L_14_ specimens had strong local abnormal high-temperature zones, which differed from the point-to-surface evolution process of the temperature field of the target coal sample, and their thermal response consistency with the target coal sample was relatively weak.

(1) Range analysis of temperature rise parameters

**Figure 8:**
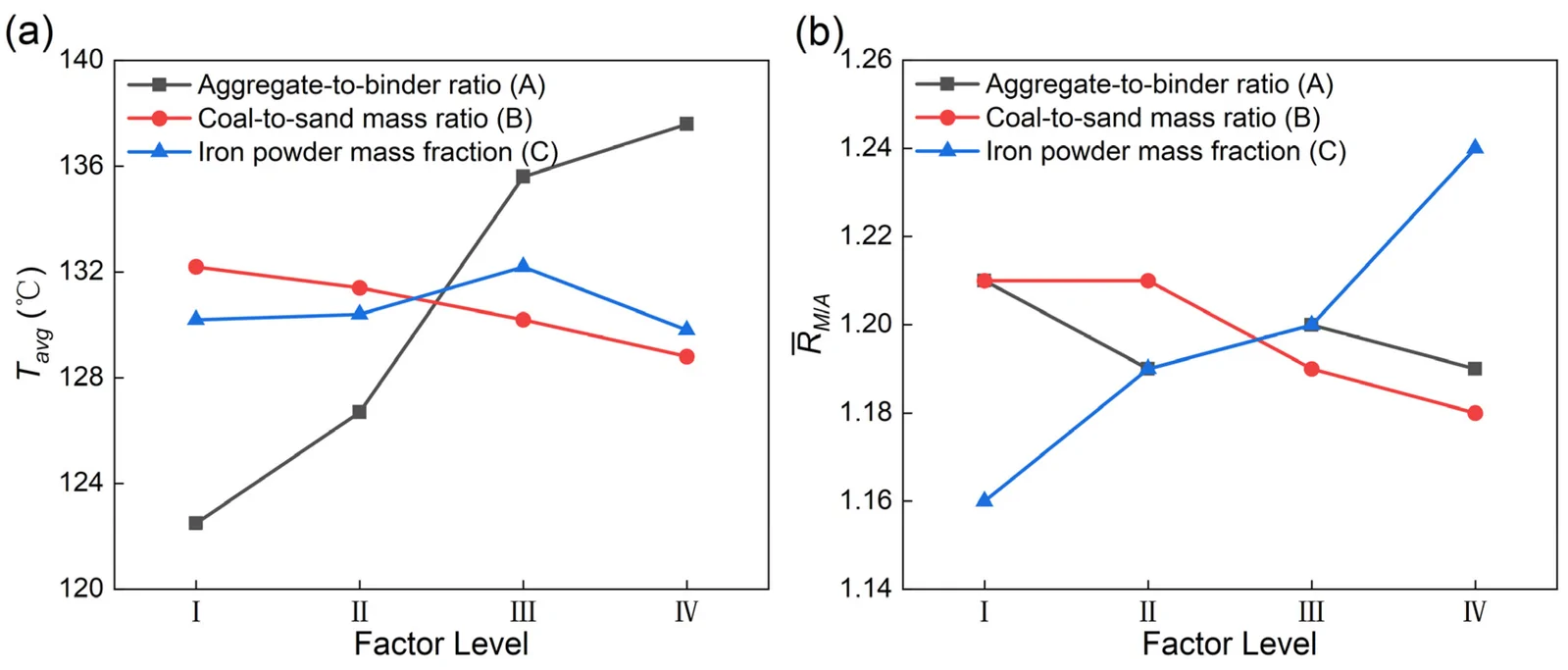
Sensitivity curves of the factors affecting the microwave heating temperature-rise parameters of coal-like specimens: (**a**) average temperature; (**b**) temperature non-uniformity coefficient.

Based on the test results of the temperature rise parameters of the specimens under microwave irradiation in Table 6, the ranges of the average temperature and the temperature non-uniformity coefficient under different factors and levels were calculated, as shown in Figure 8. The ranges of the average temperature of the specimens under factors A, B, and C are 15.1, 3.4, and 2.4, respectively, and the order of influence of each factor on the average temperature is *R*_A_ > *R*_B_ > *R*_C_, indicating that the aggregate-binder ratio (A) is the most significant factor affecting the average temperature, followed by the coal-to-sand mass ratio (B), while the iron powder mass fraction (C) has the weakest influence. As the aggregate-binder ratio (A) decreases, the average temperature of the specimen gradually increases from 122.5 °C to 137.6 °C, showing an obvious overall upward trend. However, as the coal-to-sand mass ratio (B) decreases, the average temperature of the specimen generally shows a downward trend, indicating that a higher coal powder proportion is more conducive to improving the overall temperature rise level of the specimen, but its effect is weaker than that of the aggregate-binder ratio. As the iron powder mass fraction (C) increases, the average temperature of the specimen shows only a weak fluctuation, with a slight increase at 1.0%.

Figure 8b shows the range results of the temperature non-uniformity coefficient of the specimens under different factors and levels. The ranges of the temperature non-uniformity coefficient of the specimens under factors A, B, and C are 0.02, 0.03, and 0.08, respectively. The most significant factor affecting the temperature non-uniformity coefficient is the iron powder mass fraction, followed by the coal-to-sand mass ratio, while the aggregate-binder ratio has the weakest influence. As the iron powder mass fraction increases, the temperature non-uniformity coefficient of the specimen gradually increases from 1.16 to 1.24, showing an overall upward trend, which indicates that the non-uniformity of the overall temperature field increases with the increase in the iron powder mass fraction. As the aggregate-binder ratio decreases, the temperature non-uniformity coefficient of the specimen shows only a weak fluctuation. As the coal-to-sand mass ratio decreases, the temperature non-uniformity coefficient gradually decreases, showing an overall downward trend.

(2) Variance analysis of temperature rise parameters

Variance analysis was performed on the average temperature and the temperature non-uniformity coefficient of the specimens under microwave irradiation, and the results are shown in Table 8. The significance level P of the aggregate-binder ratio (A) on the average temperature is <0.001, far less than 0.05, indicating that the aggregate-binder ratio has an extremely significant influence on the average temperature, whereas the significance levels of the coal-to-sand mass ratio (B) and the iron powder mass fraction (C) are 0.344 and 0.592, respectively, both greater than 0.05, indicating that neither has a significant influence on the average temperature.

For the temperature non-uniformity coefficient, the P-values of factors A, B, and C are 0.014, <0.001, and <0.001, respectively, indicating that both the coal-to-sand mass ratio and the iron powder mass fraction have an extremely significant influence on the temperature non-uniformity coefficient. The iron powder mass fraction directly affects the absorption and loss of microwave energy and the local heat accumulation process of the specimen, and therefore has the most significant influence on the temperature non-uniformity coefficient; the aggregate-binder ratio, on the other hand, affects the compactness of the overall structure of the coal mass, thereby influencing the heat conduction from the high-temperature zone to the low-temperature zone.

#### 4.1.3. Mechanical Similarity

**Figure 9:**
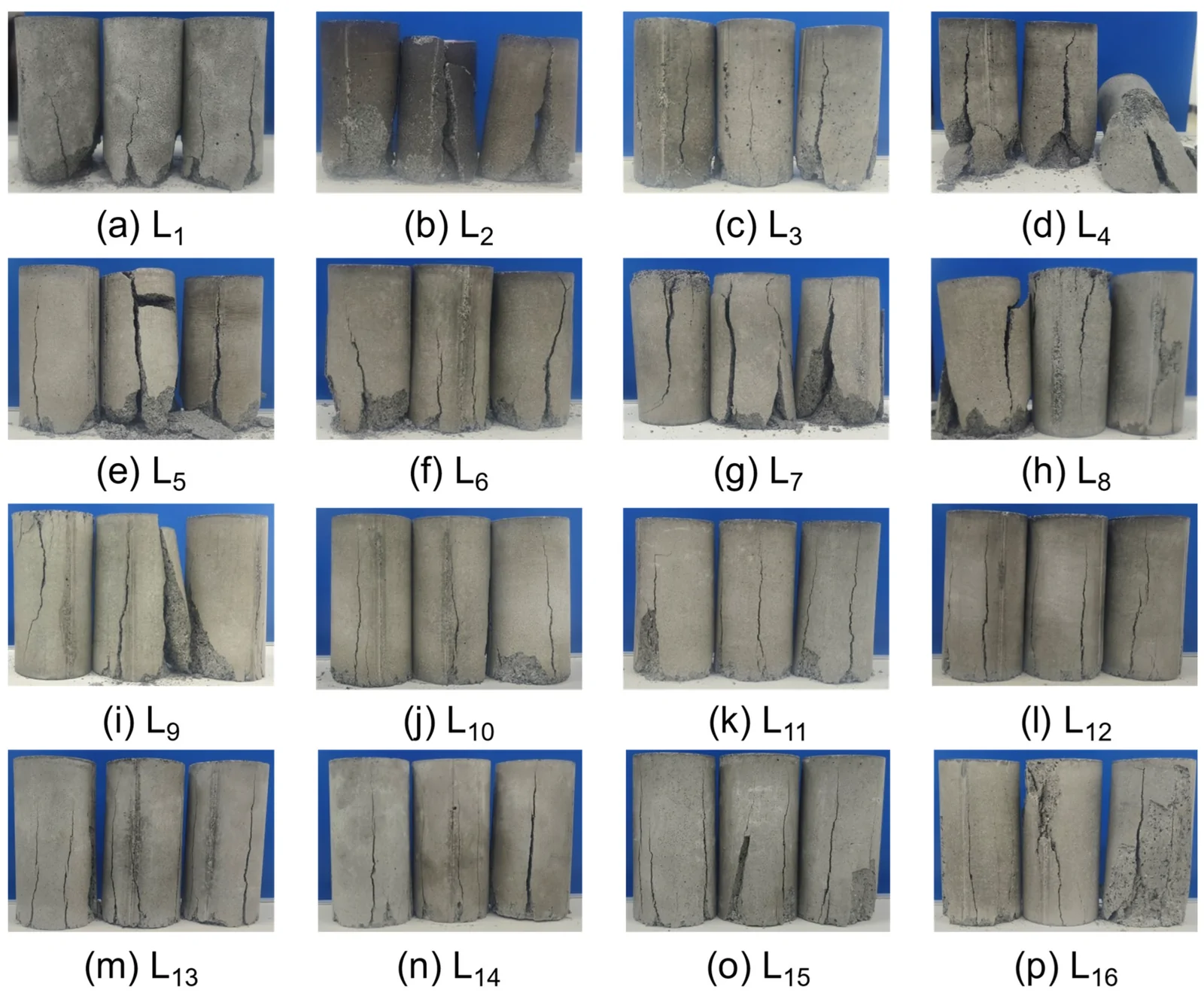
Failure patterns of specimens with different mix proportions after uniaxial compression: (**a**–**p**) failure patterns of each specimen (L_1_–L_16_).

To study the similarity between the coal-like specimens and the target coal sample in terms of mechanical properties, uniaxial compression tests were carried out on the prepared specimens to obtain the uniaxial compressive strength and elastic modulus of each specimen, and the macroscopic failure morphology of each specimen is shown in Figure 9. The failure modes of the 16 groups of specimens under uniaxial compression were generally highly consistent, all dominated by axial splitting failure, with obvious longitudinal main cracks developing along the loading direction on the specimen surface. This failure characteristic is relatively close to the splitting failure morphology of the target coal sample under uniaxial compression, indicating that the prepared coal-like specimens are consistent with the target coal sample in terms of macroscopic failure mode.

**Figure 10:**
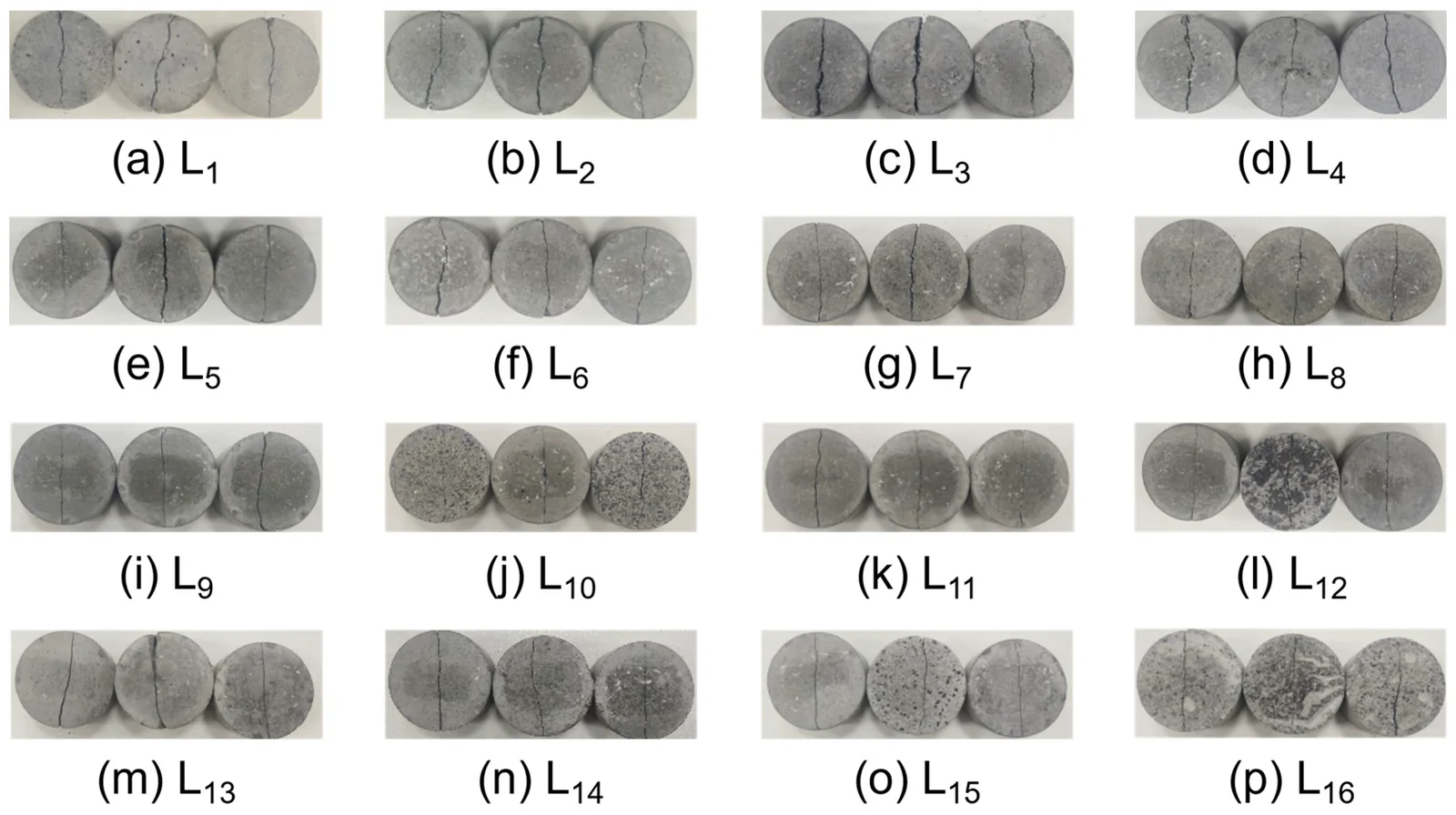
Failure patterns of specimens with different mix proportions after Brazilian splitting: (**a**–**p**) failure patterns of each specimen (L_1_–L_16_).

In this study, the tensile strength of the specimens was obtained through Brazilian splitting tests, and the macroscopic failure morphology of the specimens under splitting load was observed and analyzed, as shown in Figure 10. The failure modes of the 16 groups of coal-like specimens under Brazilian splitting were relatively consistent, all exhibiting a typical radial tensile splitting failure characteristic. Most specimens formed obvious main cracks along the loading direction, and this failure morphology is relatively close to the typical tensile failure mode of coal samples in Brazilian splitting tests.

(1) Range analysis of mechanical parameters

**Figure 11:**
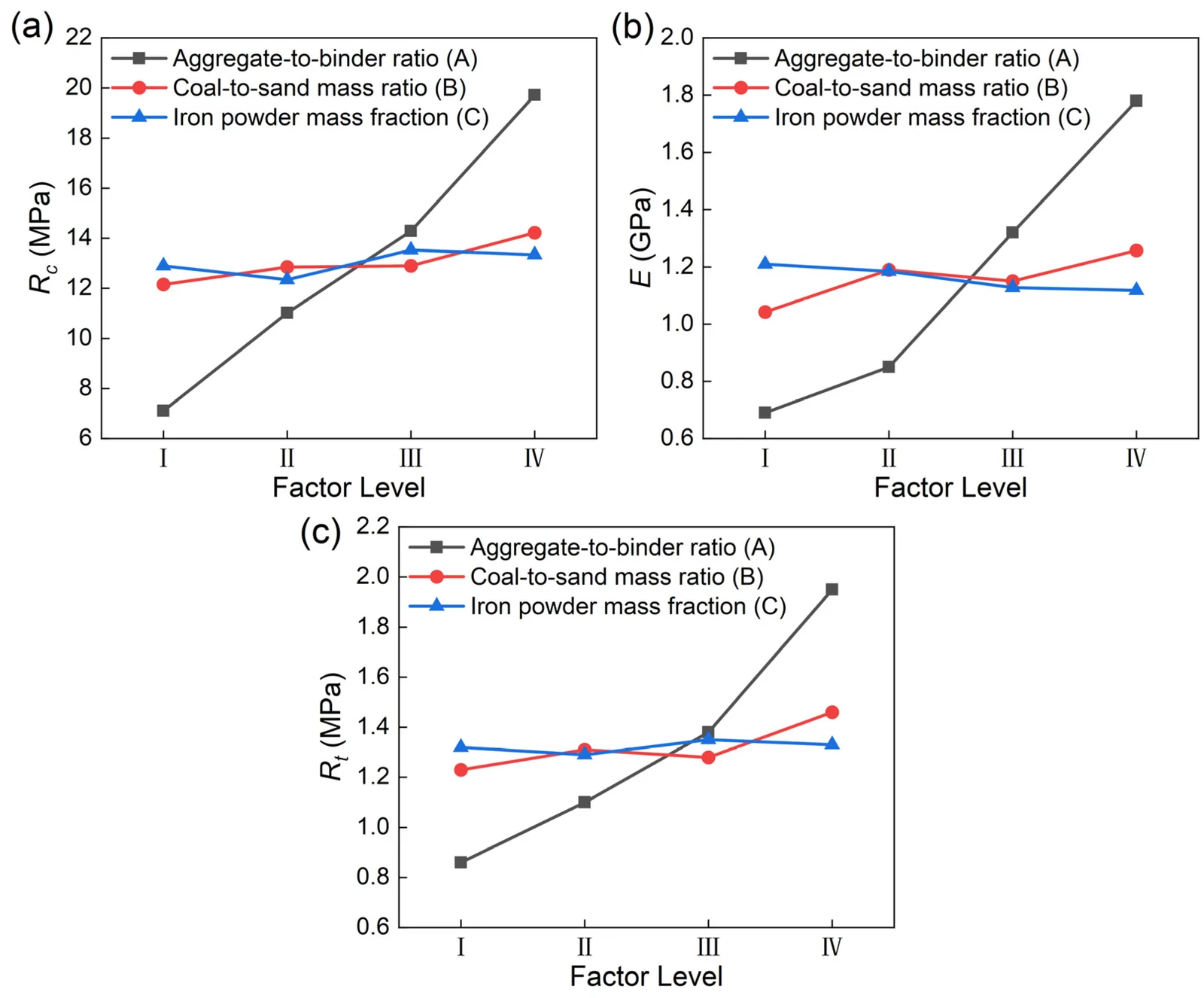
Sensitivity curves of the factors affecting the mechanical parameters of coal-like specimens: (**a**) uniaxial compressive strength; (**b**) elastic modulus; (**c**) tensile strength.

Based on the test results of the mechanical parameters of the coal-like specimens in Table 6, the mean values and ranges of the uniaxial compressive strength, elastic modulus, and tensile strength under different factors and levels were calculated, as shown in Figure 11.

Under factors A, B, and C, the ranges of the uniaxial compressive strength of the specimens are *R*_A_ = 12.61 MPa, *R*_B_ = 2.07 MPa, and *R*_C_ = 1.19 MPa; the ranges of the elastic modulus are *R*_A_ = 1.09 GPa, *R*_B_ = 0.22 GPa, and *R*_C_ = 0.09 GPa; and the ranges of the tensile strength are *R*_A_ = 1.09 MPa, *R*_B_ = 0.23 MPa, and *R*_C_ = 0.06 MPa, all showing *R*_A_ > *R*_B_ > *R*_C_. As the aggregate-binder ratio decreases, the uniaxial compressive strength of the specimen gradually increases from 7.11 MPa to 19.71 MPa, the elastic modulus from 0.69 GPa to 1.78 GPa, and the tensile strength from 0.86 MPa to 1.95 MPa, all showing an overall upward trend; a decrease in the coal-to-sand mass ratio causes the compressive strength, elastic modulus, and tensile strength to increase overall, but the influence is significantly weaker than that of the aggregate-binder ratio; the iron powder mass fraction shows only a weak fluctuation.

(2) Variance analysis of mechanical parameters

For the uniaxial compressive strength, the significance levels P of the aggregate-binder ratio (A) and the coal-to-sand mass ratio (B) are <0.05, indicating significant influences on the uniaxial compressive strength, among which the aggregate-binder ratio reaches an extremely significant level; the P-value of the iron powder mass fraction (C) is 0.240, indicating an insignificant influence. For the elastic modulus, the P-value of the aggregate-binder ratio (A) is <0.001, the P-value of the coal-to-sand mass ratio (B) is 0.040, and the iron powder mass fraction has an insignificant influence. For the tensile strength, only the aggregate-binder ratio (A) has an extremely significant influence with P < 0.001, while the influences of the coal-to-sand mass ratio (P = 0.108) and the iron powder mass fraction (P = 0.896) are both insignificant, as shown in Table 9.

### 4.2. Linear Regression Analysis of the Core Indicators

#### 4.2.1. Regression Analysis of the Imaginary Part of Complex Permittivity

To obtain the relationship between the imaginary part of the complex permittivity and each factor, the imaginary part of the complex permittivity was set as the dependent variable Y_1_, and the aggregate-binder ratio, the coal-to-sand mass ratio, and the iron powder mass fraction were set as the independent variables X_1_, X_2_, and X_3_, respectively, for multiple linear regression analysis. The regression analysis results showed that the aggregate-binder ratio and the coal-to-sand mass ratio had significant influences on the imaginary part of the complex permittivity of the similar material, whereas the iron powder mass fraction had no significant influence. Therefore, the insignificant variable X3 was removed, and linear regression analysis was performed only on the aggregate-binder ratio and the coal-to-sand mass ratio. The optimized regression model was overall significant (F = 34.690, P < 0.001), and the constant term and each regression coefficient also reached a significant level (P < 0.05). The regression coefficient results are shown in Table 10.

From Table 10, the regression coefficient of the aggregate-binder ratio is negative, indicating that the imaginary part of the complex permittivity tends to decrease as the aggregate-binder ratio increases, whereas the coal-to-sand mass ratio shows the opposite trend. The absolute value of the standardized coefficient of the coal-to-sand mass ratio (0.813) is greater than that of the aggregate-binder ratio (0.427), indicating that the coal-to-sand mass ratio has a stronger influence on the imaginary part of the complex permittivity than the aggregate-binder ratio, and is the dominant factor controlling the variation of this indicator. Finally, the regression equation of the imaginary part of the complex permittivity of the similar material can be obtained as:

*Y*_1_ = 0.296 − 0.027*X*_1_ + 0.02*X*_2_ (4)

where *Y*_1_ is the imaginary part of the complex permittivity, *X*_1_ is the aggregate-binder ratio, and *X*_2_ is the coal-to-sand mass ratio. The adjusted R^2^ of this regression model is 0.818, indicating a good fitting effect.

#### 4.2.2. Regression Analysis of the Temperature Non-Uniformity Coefficient

Taking the temperature non-uniformity coefficient as the dependent variable Y_2_, and the aggregate-binder ratio, the coal-to-sand mass ratio, and the iron powder mass fraction as the independent variables X_1_, X_2_, and X_3_, respectively, a multiple linear regression model containing three factors was established. The results showed that the coefficients of the coal-to-sand mass ratio and the iron powder mass fraction were significant, whereas the significance of the coefficient of the aggregate-binder ratio was 0.064, which did not reach the significance level. Therefore, only the significant factors, namely the coal-to-sand mass ratio and the iron powder mass fraction, were retained in the final regression model of the temperature non-uniformity coefficient, and linear regression analysis was performed again with these two factors. The optimized regression model was overall significant (F = 43.483, P < 0.001), and the constant term and each regression coefficient also reached a significant level (P < 0.05). The regression coefficient results are shown in Table 11.

From Table 11, the regression coefficients of the coal-to-sand mass ratio and the iron powder mass fraction are both positive, indicating that, with other conditions unchanged, the temperature non-uniformity coefficient tends to increase as the coal-to-sand mass ratio or the iron powder mass fraction increases. The absolute value of the standardized coefficient of the iron powder mass fraction (0.871) is greater than that of the coal-to-sand mass ratio (0.333), indicating that the iron powder mass fraction is the dominant factor controlling the variation of the temperature non-uniformity coefficient. Finally, the regression equation of the temperature non-uniformity coefficient of the similar material can be obtained as:

*Y*_2_ = 1.150 + 0.008*X*_2_ + 3.714*X*_3_ (5)

where *Y*_2_ is the temperature non-uniformity coefficient, *X*_2_ is the coal-to-sand mass ratio, and *X*_3_ is the iron powder mass fraction. The adjusted R^2^ of this model is 0.850.

#### 4.2.3. Regression Analysis of the Uniaxial Compressive Strength

Taking the uniaxial compressive strength as the dependent variable Y_3_, and the aggregate-binder ratio, the coal-to-sand mass ratio, and the iron powder mass fraction as the independent variables X_1_, X_2_, and X_3_, respectively, a multiple linear regression model containing three factors was established. The results showed that the coefficient of the aggregate-binder ratio was significant, whereas neither the coal-to-sand mass ratio nor the iron powder mass fraction reached a significant level. This indicates that, within the range of this experiment, the uniaxial compressive strength of the specimen is mainly controlled by the aggregate-binder ratio, while the direct linear influences of the coal-to-sand mass ratio and the iron powder mass fraction on the uniaxial compressive strength are weak. Therefore, only the significant factor, namely the aggregate-binder ratio, was retained in the final regression model of the uniaxial compressive strength. The regression coefficient results are shown in Table 12.

From Table 12, the regression coefficient of the aggregate-binder ratio is negative, indicating that, with other conditions unchanged, the uniaxial compressive strength tends to decrease as the aggregate-binder ratio increases. Finally, the regression equation of the uniaxial compressive strength of the similar material can be obtained as:

*Y*_3_ = 25.19 − 8.97*X*_1_ (6)

where *Y*_3_ is the uniaxial compressive strength of the specimen, and *X*_1_ is the aggregate-binder ratio. The adjusted R^2^ of this model is 0.914.

#### 4.2.4. Regression Analysis of the Elastic Modulus

Taking the elastic modulus as the dependent variable Y_4_, and the aggregate-binder ratio, the coal-to-sand mass ratio, and the iron powder mass fraction as the independent variables X_1_, X_2_, and X_3_, respectively, a multiple linear regression model containing three factors was established. The results showed that the coefficient of the aggregate-binder ratio was significant, whereas neither the coal-to-sand mass ratio nor the iron powder mass fraction reached a significant level. Therefore, only the significant factor, namely the aggregate-binder ratio, was retained in the final regression model of the elastic modulus. The regression coefficient results are shown in Table 13.

From Table 13, the regression coefficient of the aggregate-binder ratio is negative, indicating that, with other conditions unchanged, the elastic modulus tends to decrease as the aggregate-binder ratio increases. Finally, the regression equation of the elastic modulus of the similar material can be obtained as:

*Y*_4_ = 2.212 − 0.815*X*_1_ (7)

where *Y*_4_ is the elastic modulus of the specimen, and *X*_1_ is the aggregate-binder ratio. The adjusted R^2^ of this model is 0.876.

### 4.3. Determination and Verification of the Optimal Mix Proportion

Taking the parameters of the target coal sample as the constraints, the optimal mix proportion of the similar material was solved using the linear regression equations of each core indicator under a permissible error of 10%. Since the four indicators have inconsistent influence laws on the mix proportion, there is no unique analytical solution that can make all core indicators simultaneously and accurately reach the target values; instead, only a mix proportion with a relatively good comprehensive matching effect can be obtained within the permissible error range.

By simultaneously solving and checking the regression Equations (4)–(7), the optimal mix proportion of each material was obtained, namely an aggregate-binder ratio of 1.45:1, a coal-to-sand mass ratio of 4.11:1, and an iron powder mass fraction of 0.73%. Considering the operational convenience and implement ability of the mix proportion in the actual specimen preparation process, it was further modified to an aggregate-binder ratio of 1.45:1, a coal-to-sand mass ratio of 4:1, and an iron powder mass fraction of 0.75%. After substituting this mix proportion into the four core indicator regression equations, the imaginary part of the complex permittivity, the temperature non-uniformity coefficient, the compressive strength, and the elastic modulus were all within the permissible error range of 10% relative to the target values, indicating that this mix proportion can well balance the similarity requirements in terms of dielectric properties, microwave-heating temperature rise characteristics, and mechanical properties.

**Figure 12:**
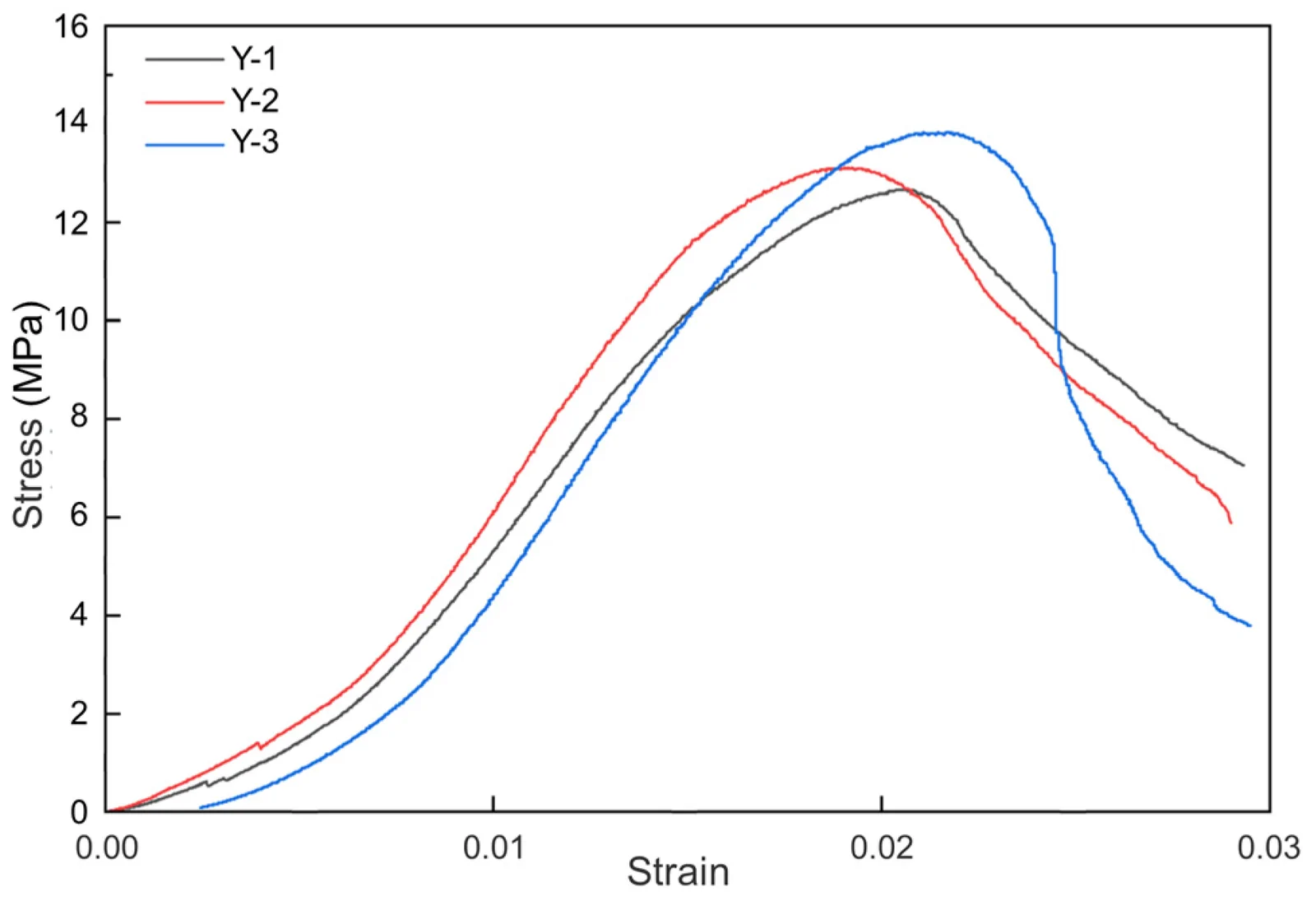
Stress-strain curves of the specimens with the optimal mix proportion.

**Figure 13:**
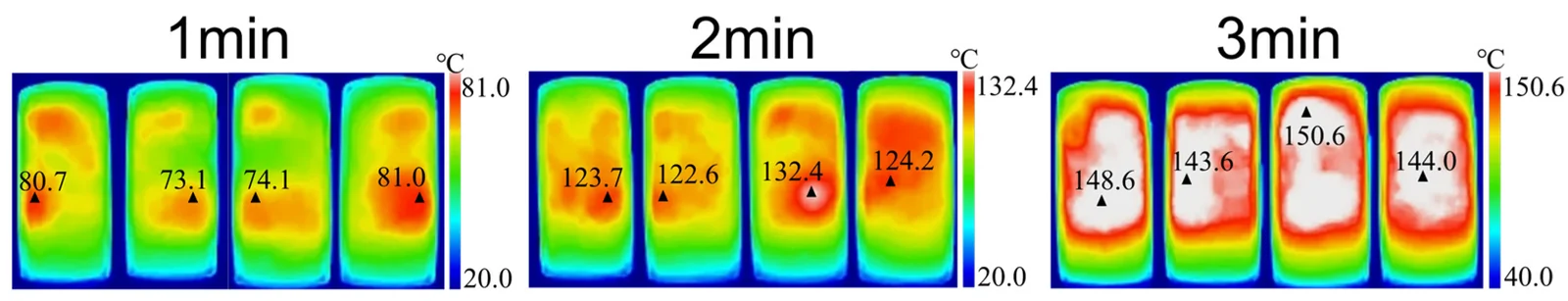
Infrared thermal images of the optimal-mix specimen under microwave irradiation.

According to the above mix proportion, a new batch of standard specimens with a diameter of 50 mm and a height of 100 mm, as well as a diameter of 50 mm and a height of 25 mm, was prepared to carry out relevant experiments. The uniaxial compressive stress-strain curves and infrared thermal images are shown in Figure 12 and Figure 13, respectively, to verify the feasibility of the mix proportion.

From Table 14, the deviations of the real and imaginary parts of the complex permittivity from the target values are small, indicating that the specimen has good matching performance in terms of dielectric response. The average temperature is slightly lower than the target value, whereas the temperature non-uniformity coefficient is basically consistent with the target value, indicating that the specimen can not only reach an overall temperature rise level close to that of the target coal sample but also reproduce the temperature non-uniformity of the coal sample under microwave action well. The compressive strength is relatively close to the target value, the elastic modulus is slightly higher, and the tensile strength is slightly lower, but all remain within a relatively close range. As defined in Section 2.3, the tensile strength is treated only as a verification index rather than a core optimization constraint, and it was therefore not included in the 10% optimization constraints. In the verification test, the relative error of the tensile strength is −6.02%, which lies within the stated 10% criterion, so the tensile strength of the optimal-mix material is acceptable as a verification parameter. Among the four core indicators, the imaginary part of the complex permittivity, the temperature non-uniformity coefficient, and the compressive strength are all within 10%, whereas the elastic modulus shows a relative error of +11.46%, which slightly exceeds the 10% criterion. This small exceedance arises mainly because the elastic modulus is more sensitive to the internal microstructure and microcracks of the specimens than the compressive strength and is therefore more difficult to control precisely during specimen preparation. Nevertheless, an error of 11.46% is only marginally above the 10% threshold and the elastic modulus remains of the same order as the target value, so this exceedance does not materially affect the overall similarity of the material.

Overall, the coal-like specimens prepared according to the optimal mix proportion show good consistency with the target coal sample in terms of dielectric properties, microwave-heating temperature rise characteristics, and mechanical properties, indicating that this mix proportion can well characterize the microwave thermal response and mechanical characteristics of the target coal sample.

## 5. Discussion

### 5.1. Quantitative Comparison and Physical Mechanisms

Existing coal-like materials have been developed primarily for coal-and-gas-outburst simulation, reproducing the mechanical, deformation, and gas-flow (permeability) characteristics of coal but not the microwave dielectric and thermal responses [42]. In contrast, the material developed in this study simultaneously matches the target coal in its dielectric, thermal, and mechanical responses. As summarized in Table 14, the real and imaginary parts of the complex permittivity of the optimal-mix material are 3.659 and 0.355, close to the target values of 3.474 and 0.336 (relative errors of +5.33% and +5.65%); the average temperature and the temperature non-uniformity coefficient are 120.2 °C and 1.22, close to the target values of 123.4 °C and 1.21 (errors of −2.59% and +0.83%); and the uniaxial compressive strength and the elastic modulus are 13.20 MPa and 1.07 GPa, close to the target values of 13.41 MPa and 0.96 GPa (errors of −1.57% and +11.46%). For comparison, the uniaxial compressive strength of the coal-like (similar) materials reported in the literature for hydraulic-fracturing or general physical simulation is only 2.84–7.16 MPa with an elastic modulus of 0.142–0.515 GPa [43], or about 3.4–3.9 MPa [44], considerably lower than the target-coal strength of 13.41 MPa. By contrast, the optimal-mix material developed here attains a compressive strength of 13.20 MPa and an elastic modulus of 1.07 GPa, demonstrating the wide-range adjustability of the mechanical parameters through the rational design of the aggregate-to-binder ratio. This simultaneous quantitative agreement in the three coupled aspects—dielectric, thermal, and mechanical—distinguishes the present material from earlier coal-like materials that reproduce only the mechanical and seepage characteristics of coal [42]. This agreement is achieved through three dominant physical mechanisms. First, the dielectric response is governed by the coal powder content: as the main dielectric-loss component, coal powder converts microwave energy into heat through the dipolar and interfacial polarization of its polar functional groups and conductive minerals under the 2.45 GHz microwave field, so the coal-to-sand mass ratio controls the imaginary part of the complex permittivity. Second, the non-uniform temperature rise originates from the selective microwave absorption of the iron powder particles, which act as localized microwave absorbers and generate hot spots and steep temperature gradients; heat is then conducted from these hot spots to the surrounding cooler regions, producing temperature gradients that drive thermal-stress generation and fracture initiation, so the iron powder mass fraction dominates the temperature non-uniformity coefficient. Third, the mechanical behavior is mainly controlled by the aggregate-to-binder ratio, because the cement hydration products bind the coal-powder and river-sand aggregate particles and determine the strength and stiffness of the material; specifically, the compressive strength is governed by cementation and frictional interlocking between aggregate particles, whereas the tensile strength is controlled mainly by the bonding strength at the aggregate–binder interfaces and by internal microcracks. These mechanisms are consistent with the sensitivity and regression analyses in Section 4.1 and Section 4.2.

### 5.2. Practical Applications and Limitations

The optimized coal-like material can support repeatable, large-scale open-ended microwave borehole fracturing experiments, because its raw materials are inexpensive and widely available and its preparation process is simple and stable, which overcomes the sampling, transportation, and repeatability difficulties of large-sized natural coal. In such experiments, the material can be used to investigate crack initiation, crack propagation, and permeability enhancement around a microwave-heated borehole under controlled and reproducible conditions. Nevertheless, several limitations should be acknowledged. The present validation was conducted under unstressed, dry, room-temperature conditions and for a single bituminous coal; the material has not yet been validated under in-situ stress, varying moisture contents, or different coal types. Therefore, the direct extrapolation of the present results to field applications requires further validation under these conditions.

In addition, the present similarity evaluation is based on macroscopic indicators—namely, the complex permittivity, the temperature-rise parameters, and the mechanical parameters—whereas the chemical composition of the target coal and the microstructural (textural) evolution of coal after microwave irradiation were not characterized by EDS or SEM/TEM; these characterizations will be incorporated in future work on the relationship between mineral composition and dielectric properties. Furthermore, the temperature-rise characteristics were established under a fixed reference condition (1 kW for 3 min), and the additive-screening tests were deliberately conducted at a fixed power to isolate the additive effect, so the dependence of the temperature rise on the microwave power remains to be systematically investigated.

## 6. Conclusions

(1) A similarity evaluation system for coal-like materials covering the complex dielectric properties, microwave non-uniform temperature rise characteristics, and mechanical properties was established, in which the imaginary part of the complex permittivity, the temperature non-uniformity coefficient under microwave irradiation, the uniaxial compressive strength, and the elastic modulus were taken as the core evaluation indicators, while the real part of the complex permittivity, the average temperature under microwave irradiation, and the tensile strength were taken as the verification indicators.

(2) Based on multiple linear regression, the regression relationships between the imaginary part of the complex permittivity, the temperature non-uniformity coefficient, the uniaxial compressive strength, and the elastic modulus and the material mix proportion parameters were established. Under the condition that the error of each core indicator relative to the target coal sample is controlled within 10%, the optimal mix proportion was determined as: an aggregate-binder ratio of 1.45:1, a coal-to-sand mass ratio of 4:1, and an iron powder mass fraction of 0.75%.

(3) The coal-like specimens prepared according to the optimal mix proportion show good consistency with the target coal sample in terms of complex dielectric properties, microwave temperature rise characteristics, and mechanical properties, and this mix proportion can well characterize the microwave thermal response and mechanical characteristics of the target coal sample. Overall, the integrated similarity design—simultaneously reproducing the dielectric, thermal, and mechanical responses of the target coal within a single material—is the key contribution of this work and provides a reliable basis for large-scale open-ended microwave borehole fracturing experiments.

## Figures and Tables

**Table 1:** Temperature parameters of the target coal sample after 3 min of microwave irradiation.

Microwave Power	*T_max_*/°C	${\bar{T}}_{\max}$/°C	*T_avg_*/°C	${\bar{T}}_{\mathrm{avg}}$/°C	*R_M_*_/*A*_	${\bar{R}}_{M/A}$
1 kW	150.8	149.6	125.1	123.4	1.21	1.215
	148.6		122.8		1.21	
	150.2		121.2		1.24	
	148.9		124.4		1.20	

**Table 2:** Mechanical parameters of the target coal sample.

Sample	Size	*R_c_*/MPa		*E*/GPa		*R_t_*/MPa	
A1	50 mm × 100 mm	13.01	13.41	0.96	0.96	-	-
A2		12.40		0.85		-	-
A3		14.82		1.06		-	-
B1	50 mm × 25 mm	-	-	-	-	1.26	1.33
B2		-	-	-	-	1.32	
B3		-	-	-	-	1.41	

**Table 3:** Test scheme for the temperature-rise characteristics of coal powder-additive mixtures.

Scheme	Microwave Frequency	Microwave Power	Test Time/min	Coal Powder Mass Fraction	Additive Mass Fraction
A	2.45 GHz	1 kW	3 min	98%	2%
B	2.45 GHz	1 kW	3 min	96%	4%
C	2.45 GHz	2 kW	3 min	98%	2%
D	2.45 GHz	2 kW	3 min	96%	4%

**Table 4:** Factor levels of the orthogonal array.

Level	A (m_aggregate_:m_binder_)	B (m_coal powder_:m_river sand_)	C (m_iron powder_)
I	2:1	4:1	0%
II	3:2	2:1	0.5%
III	1:1	1:1	1.0%
IV	2:3	1:2	2.0%

**Table 5:** Design of the orthogonal test scheme.

No.	A (m_aggregate_:m_binder_)	B (m_coal powder_:m_river sand_)	C (m_iron powder_)
L_1_	2:1	4:1	0%
L_2_	2:1	2:1	0.5%
L_3_	2:1	1:1	1.0%
L_4_	2:1	1:2	2.0%
L_5_	3:2	4:1	0.5%
L_6_	3:2	2:1	1.0%
L_7_	3:2	1:1	2.0%
L_8_	3:2	1:2	0%
L_9_	1:1	4:1	1.0%
L_10_	1:1	2:1	2.0%
L_11_	1:1	1:1	0%
L_12_	1:1	1:2	0.5%
L_13_	2:3	4:1	2.0%
L_14_	2:3	2:1	0%
L_15_	2:3	1:1	0.5%
L_16_	2:3	1:2	1.0%

**Table 6:** Physical properties of the coal-like materials.

Sample	*ε*′	*ε*″	${\bar{T}}_{\max}$/°C	${\bar{T}}_{\mathrm{avg}}$°C	${\bar{R}}_{M/A}$	*R_c_*/MPa	*E*/GPa	*R_t_*/MPa
L_1_	3.548	0.319	146.6	123.4	1.19	7.55	0.58	0.69
L_2_	3.510	0.298	145.2	120.1	1.21	8.68	0.73	0.89
L_3_	3.454	0.268	150.0	124.7	1.20	8.63	0.63	0.78
L_4_	3.461	0.254	150.9	121.9	1.24	9.88	0.82	1.07
L_5_	3.617	0.323	156.6	130.8	1.20	11.23	0.77	0.97
L_6_	3.586	0.304	156.0	128.6	1.21	10.79	0.88	1.08
L_7_	3.623	0.257	151.9	124.9	1.22	11.51	0.82	1.00
L_8_	3.478	0.245	139.3	122.6	1.14	12.54	0.93	1.33
L_9_	3.734	0.351	166.5	138.4	1.20	14.80	1.20	1.41
L_10_	3.765	0.315	169.7	136.1	1.25	13.87	1.21	1.39
L_11_	3.659	0.290	154.9	134.4	1.15	14.37	1.39	1.37
L_12_	3.664	0.264	157.7	133.7	1.18	14.54	1.48	1.33
L_13_	3.814	0.340	181.6	136.1	1.25	18.12	1.62	1.84
L_14_	3.841	0.345	164.9	140.6	1.17	18.13	1.94	1.89
L_15_	3.825	0.306	160.4	136.8	1.17	17.84	1.76	1.95
L_16_	3.816	0.286	161.4	136.9	1.18	20.76	1.80	2.11

**Table 7:** Analysis of variance of the real and imaginary parts of complex permittivity.

Complex Permittivity	Source	Sum of Squares	Degrees of Freedom	Mean Square	F	P
*ε*′	A	0.254	3	0.085	44.756	<0.001
	B	0.014	3	0.005	2.535	0.153
	C	0.002	3	0.001	0.432	0.738
	Error	0.011	6	0.002	/	/
	Total	0.282	15	/	/	/
*ε* ^″^	A	0.004	3	0.001	18.036	0.002
	B	0.013	3	0.004	61.322	<0.001
	C	0.000	3	0.000	1.235	0.376
	Error	0.000	6	0.000	/	/
	Total	0.017	15	/	/	/

**Table 8:** Analysis of variance of average temperature and temperature non-uniformity coefficient.

Temperature	Source	Sum of Squares	Degrees of Freedom	Mean Square	F	P
*T_avg_*	A	619.120	3	206.373	32.140	<0.001
	B	25.989	3	8.663	1.349	0.344
	C	13.247	3	4.416	0.688	0.592
	Error	38.526	6	6.421	/	/
	Total	696.883	15	/	/	/
*R_M_*_/*A*_	A	0.001	3	0.000	8.500	0.014
	B	0.002	3	0.001	25.000	<0.001
	C	0.012	3	0.004	123.500	<0.001
	Error	0.000	6	0.000	/	/
	Total	0.016	15	/	/	/

**Table 9:** Analysis of variance of the mechanical parameters of coal-like materials.

Mechanical Parameters	Source	Sum of Squares	Degrees of Freedom	Mean Square	F	P
Uniaxial compressive strength	A	341.428	3	113.809	187.005	<0.001
	B	8.914	3	2.971	4.882	0.047
	C	3.364	3	1.121	1.843	0.240
	Error	3.652	6	0.609	/	/
	Total	357.358	15	/	/	/
Elastic modulus	A	2.908	3	0.969	158.907	<0.001
	B	0.097	3	0.032	5.314	0.040
	C	0.024	3	0.008	1.309	0.355
	Error	0.037	6	0.006	/	/
	Total	3.066	15	/	/	/
Tensile strength	A	2.654	3	0.882	68.931	<0.001
	B	0.121	3	0.040	3.151	0.108
	C	0.007	3	0.002	0.195	0.896
	Error	0.077	6	0.013	/	/
	Total	2.850	15	/	/	/

**Table 10:** Regression coefficients of the regression equation for the imaginary part of complex permittivity.

Model	Unstandardized Coefficients		Standardized Coefficients	t	Significance
	B	Std. Error	Beta		
Constant	0.296	0.011	/	26.896	<0.001
Aggregate-to-binder ratio (X_1_)	−0.027	0.007	−0.427	−3.872	0.002
Coal-to-sand mass ratio (X_2_)	0.020	0.003	0.813	7.375	<0.001

**Table 11:** Regression coefficients of the regression equation for temperature non-uniformity coefficient.

Model	Unstandardized Coefficients		Standardized Coefficients	*t*	Significance
	B	Std. Error	Beta		
Constant	1.150	0.007		174.799	<0.001
Coal-to-sand mass ratio (X_2_)	0.008	0.002	0.333	3.327	0.005
Iron powder mass fraction (X_3_)	3.714	0.426	0.871	8.712	<0.001

**Table 12:** Regression coefficients of the regression equation for uniaxial compressive strength.

Model	Unstandardized Coefficients		Standardized Coefficients	*t*	Significance
	B	Std. Error	Beta		
Constant	25.190	1.097		22.964	<0.001
Aggregate-to-binder ratio (X_1_)	−8.970	0.658	−0.959	−13.635	<0.001

**Table 13:** Regression coefficients of the regression equation for elastic modulus.

Model	Unstandardized Coefficients		Standardized Coefficients	*t*	Significance
	B	Std. Error	Beta		
Constant	2.212	0.109		20.224	<0.001
Aggregate-to-binder ratio (X_1_)	−0.815	0.079	−0.940	−10.329	<0.001

**Table 14:** Test results of the optimal mix proportion.

Item	Complex Permittivity		Average Temperature	Temperature Non-Uniformity Coefficient	Compressive Strength	Elastic Modulus	Tensile Strength
	Real Part	Imaginary Part					
Target value	3.474	0.336	123.4	1.21	13.41	0.96	1.33
Measured value	3.659	0.355	120.2	1.22	13.20	1.07	1.25
Relative error	5.33%	5.65%	−2.59%	0.83%	−1.57%	11.46%	−6.02%

## Data Availability

The original contributions presented in this study are included in the article. Further inquiries can be directed to the corresponding authors.
